# Draft Genome Sequence of the Phosphate-Solubilizing Bacterium *Pseudomonas argentinensis* Strain SA190 Isolated from the Desert Plant *Indigofera argentea*

**DOI:** 10.1128/genomeA.01431-16

**Published:** 2016-12-22

**Authors:** Feras F. Lafi, Intikhab Alam, Rene Geurts, Ton Bisseling, Vladimir B. Bajic, Heribert Hirt, Maged M. Saad

**Affiliations:** a King Abdullah University of Science and Technology (KAUST), Biological and Environmental Sciences and Engineering Division (BESE), Thuwal, Kingdom of Saudi Arabia; b Computational Bioscience Research Center (CBRC), King Abdullah University of Science and Technology (KAUST), Thuwal, Kingdom of Saudi Arabia; c Department of Plant Sciences, Laboratory of Molecular Biology, Wageningen University, Wageningen, Netherlands

## Abstract

*Pseudomonas argentinensis* strain SA190 is a plant endophytic-inhabiting bacterium that was isolated from root nodules of the desert plant *Indigofera argentea* collected from the Jizan region of Saudi Arabia. Here, we report the genome sequence of SA190, highlighting several functional genes related to plant growth–promoting activity, environment adaption, and antifungal activity.

## GENOME ANNOUNCEMENT

In an effort to explore the microbial diversity of the desert pioneer plants, the Darwin21 project (http://www.darwin21.net) has been established. Under the project, extensive microbial isolations from the roots of different desert plants have been conducted. Preliminary results have revealed a large diversity of bacterial species with a potential to promote the growth of *Arabidopsis thaliana* plants under abiotic stresses. A select number of these strains were sequenced and characterized as described previously (1). Phosphate-solubilizing bacteria, particularly *Pseudomonas* spp., dominated our collection and are encountered in other plants (2–5). *P. argentinensis* strain SA190 is an endophytic bacterium that was isolated from surface-sterilized root nodules of the pioneer plant *Indigofera* *argentea* Burm.f. (*Fabaceae*). Plants were collected in the Jizan area (16°56.475′N, 42°36.694′E) of Saudi Arabia. Based on the 16S rRNA gene sequences, the strain SA190 is closely related (99% gene similarity) to *P. argentinensis* strain CH01 (NR_043115.1) and *P. argentinensis* strain PA01 (AY691189.2) (6).

The genomic DNA of strain SA190 was extracted using the Qiagen DNeasy blood and tissue kit, following the manufacturer’s protocol. The DNA was then sequenced using paired-end Illumina MiSeq reads, with the library prepared as described previously (1). Contig assembly was done with SPAdes assembler version 3.6 (7) with a 1-kb contig cutoff size. *De novo* assembly of MiSeq reads for *P. argentinensis* strain SA190 resulted in 27 contigs with a total length of 5,055,230 bp and a mean contig size of 187,230 bp. The *N*_50_ was 496,934 bp, and the *L*_50_ was reached in four contigs. The GC content of this genome was 64%. MegaBLAST (8) searches of strain SA190 concatenated genomes against the NCBI reference genome database (http://www.ncbi.nlm.nih.gov/genome) revealed that the closest relative genomes with a 78% query coverage and 96% sequence identity belonged to *P. fulva* 12-X (NC_015556.1) isolated from a rice paddy. The annotation of *P. argentinensis* SA190 was carried out using the default INDIGO pipeline (9) with the exception of open reading frame (ORF) prediction by FragGeneScan (10). The annotation of SA190 resulted in 3,661 ORFs, six rRNAs, 57 tRNAs, and 125 ncRNAs. The annotation predicted a number of genes related to growth-promotion activity, including gene-coding clusters for phosphate solubilization: six genes for pyrroloquinoline quinone synthesis (PQQ) (EC: 1.1.5.2) organized in one operon *pqq*EDCBAF; a gene (*acd*S) that encodes for 1-aminocyclopropane-1-carboxylic acid deaminase (ACC deaminase) (EC: 3.5.99.7, K01505); and a key enzyme involved in plant growth–promotion activities (11, 12). Furthermore, genes involved in antifungal activity were found, e.g., glucan endo-1,3-beta-d-glucosidase (EC 3.2.1.39) and chitinase (EC: 3.2.1.14), which has been reported to be involved in fending off plant pathogenic fungi (13). Further analysis of the genome sequence of strain SA190 will help to elucidate the metabolic pathways involved in plant growth–promoting interaction and further an understanding of molecular mechanisms for controlling some pathogens of soil-borne plant diseases.

### Accession number(s).

The genome of *P. argentinensis* SA190 was deposited at DDBJ/EMBL/GenBank under the accession number LWDZ00000000. The version described in this paper is the first version, LWDZ01000000.
